# Contractile properties of the right atrial myofilaments in patients with myxomatous mitral valve degeneration

**DOI:** 10.1186/1471-2261-14-119

**Published:** 2014-09-16

**Authors:** Constanze Bening, Uwe Mehlhorn, Lars Oliver Conzelmann, Nicole Stumpf, Anjuli Sikand, Christian-Friedrich Vahl

**Affiliations:** Department of Cardiothoracic and Vascular Surgery, Medical Centre of the Johannes Gutenberg University Mainz, Langenbeckstr.1, 55131 Mainz, Germany

**Keywords:** Calcium sensitivity, Myxomatous mitral valve degeneration, Skinned fiber

## Abstract

**Background:**

Myxomatous degeneration of the mitral valve is a common pathological finding in mitral valve surgery and the most common reason for severe mitral valve regurgitation. Considering the importance of right ventricular remodeling and global function after mitral valve surgery we tried to elucidate a possible association of myxomatous mitral valve and impairment of right atrial and ventricular function, which might have an impact on global ventricular performance after mitral valve surgery.

**Methods:**

Right atrial tissue was harvested from 47 patients undergoing mitral valve surgery. We took the trabeculae from the right auricle, which was resected at the right auricle for implementation of extracorporal circulation. The tissue was skinned and prepared in a 24 h-lasting procedure to create small fibers for hinging them in the "muscle machine", an experimental set-up, created for pCa-force measurements.

**Results:**

Patients without myxomatous mitral valve developed significantly more force (4.0 mN ± 0.8 mN) at the highest step of calcium concentration compared to 2.7 mN ± 0.4 mN in group of patients with myxomatous valve degeneration (p 0.03). Calcium sensitivity in the myxomatous valve group was at pCa 6.0 and in the non-myxomatous group at pCa 5. Furthermore we observed a significant difference in ejection fraction (EF) among the groups: 49% in the non-myxomatous group versus 57% in the myxomatous group (p 0.03). In the non-myxomatous group 5 patients had diastolic dysfunction grade I-II (22,7%), in group I 10 patients (40%). This was also significant (p 0.04).

**Conclusions:**

Patients with myxomatous mitral valve degeneration seem to have reduced force capacities. Calcium sensitivity is higher compared to the non-myxomatous group, which might be a compensatory mechanism to cover the physiological demand. Furthermore we suggest a higher incidence of diastolic dysfunction in patients with myxomatous mitral valve degeneration, which might have an impact on ventricular remodeling after mitral valve surgery.

## Background

**Figure 1:**
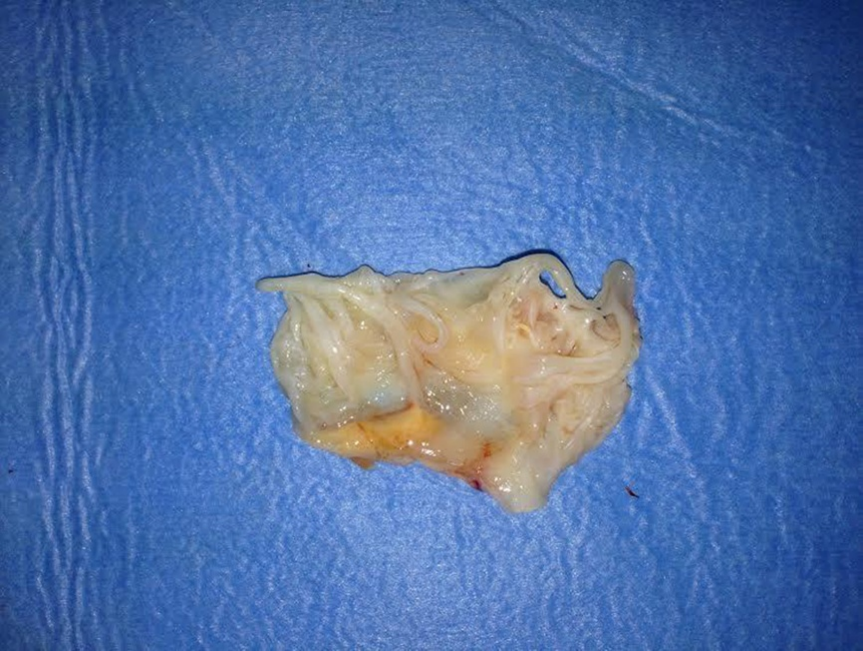
**Myxomatous Mitral Valve Leaflet.**

Myxomatous degeneration of the mitral valve is a common pathological finding in mitral valve surgery (Figure 1). The histological findings of myxomatous mitral valve degeneration (MMVD) include that mitral valve leaflets are more extensible and less stiff circumferentially and radially compared to normal mitral valves [1, 2]. Furthermore chordae are longer and leaflets are enlarged, thus failing at significantly lower tensile stress than normal chordae [2]. Additionally enlargement of the mitral annulus accompanies myxomatous valve disease. The tri-layered structure of the valve as well as the organization of the matrix structure is destroyed by accumulation of mechanically inadequate glycosaminoglycans which are increased in this tissue [3]. Further collagen fibrils change their organization structure and loose mechanical stability and flexibility [4]. This finding is not uncommon and also often found in a localized form [5]. This histological characteristic can also be associated with degenerative valvular diseases like M. Barlow, Marfan’s syndrome or fibroelastic deficiency syndrome and other connective tissue disorders but can also be found in the elderly [5] which might allow the assumption of MMVD to be a symptom of ageing of the valve.

But this finding is not new: The first description of myxomatous mitral valves can already be found in 1910 (Dewitzky, Felsenreich and von Weisner 1915). It was found to be more common in the posterior cusp, but it was thought to have no hemodynamic significance, although it was assumed, that incidence increases with age [5]. The assumption of being a sign of ageing comes from examinations with canines: a wide range of examinations were performed in dogs, because the myxomatous mitral valve degeneration (MMVD) is the most common cardiac disease in dogs and there are strong evidences for a close dependency of age and this disease [6]. Some analogies absolutely exist between humans and dogs [7].

Nevertheless some inconsistencies exist: the prevalence of MMVD in humans is estimated at 2-3% and is described to be equally distributed among men and women [8], although mitral valve prolapse affects more women than men [9]. There are some evidences in humans for the association of ageing and accompanying pathological conditions in patients with myxomatous mitral valves but the etiology and associated lesions are not clear, but issue of various assumptions [5].

Read defined the myxomatous mitral valve as the "floppy valve syndrome" and thought it to be a special entity of the Marfan’s syndrome [10, 11].

So clear definitions as well as possible genetic associations remain uncertain. However it seems obvious that patients with a myxomatous mitral valve do not show any clinical signs of impairment of cardiac function and therefore clinical relevance seems to be of academically interest, but the affection of the mechanical properties (i.e. ruptured chordae, prolapse, deformed cusps) due to myxomatous degeneration [1, 2] especially in older patients can be rapidly fatal and might influence the outcome and survival.

The issue of taking right atrial tissue, follows some intentions: There are many studies about the left ventricular and left atrial function in chronic mitral regurgitation or patients undergoing mitral valve surgery [12, 13], which show a significant impairment of LA filling, LA contraction and ejection fraction as well a latent LV dysfunction in patients with mitral regurgitation. But investigations about right ventricular and atrial function after cardiac surgery and especially mitral valve surgery are still rare. Although impaired right ventricular function after mitral valve surgery is associated with poor survival rate [14] and impairment of right ventricular function is frequent (30%) and a strong predictor of postoperative cardiovascular survival [13].

As a second clinical interest could be the assumption that the condition of possible association of myxomatous mitral valve and impairment of right atrial and/or ventricular function might have an impact on ventricular performance and remodeling after mitral valve surgery and might influence the decision of surgical repair or replacement of the valve [15].

Therefore we underwent a study to examine a potential impairment of contractile function in patients with myxomatous mitral valve undergoing valve replacement or repair.

## Methods

### Patients

We observed 47 patients undergoing mitral valve repair (MVr) or mitral valve replacement (MVR) and divided after the pathological finding of myxomatous mitral valve (see Table 1). We harvested the tissue intraoperatively and collected the pathological findings afterwards. 25 patients had a myxomatous degeneration (53%). In the myxomatous mitral valve group (group I) the mean age was 60 ± 16 years and consisted of 13 males and 12 females. 16 patients showed as leading feature mitral valve regurgitation (64%), 9 patients a mitral valve stenosis (36%) also as leading feature (Not isolated valve stenosis). The mean EF in this group was 57%. 12 patients underwent a mitral valve repair (48%) and 13 replacement of the valve (52%).

**Table 1:** **Patient’s clinical characteristics**

	Myxomatous MV	Non-Myxomatous MV	p
Age	60 ± 16 years	67 ± 13 years	n.s.
Gender	13 Males (52%)	8 Males (36%)	n.s.
	12 Females (48%)	14 Females (63%)	
Leading Valve			
Pathology	Regurgitation (64%)	Regurgitation (90%)	n.s.
	Stenosis (36%)	Stenosis (10%)	
Surgical Procedure			
Repair	48%	72.7%	0.02
Replacement	52%	36.3%	n.s.
Ejection Fraction:	57%	49%	0.03
Atrial Dilatation	36%	68%	0.01
Ventricular Dilat.	12%	13%	n.s.
Annulus Dilat.	4%	9%	n.s.
Atrial Fibrillation	24%	38%	n.s.
Valve Prolapse	36%	27%	0.02
Diastolic			
Dysfunction	40%	22.7%	0.04

The group of patients without myxomatous mitral valve (group II) consisted of 22 patients with 8 males (36%) and 14 females (63%). The mean age was 67 ± 13 years. 20 patients showed as leading echocardiographic finding a mitral regurgitation (90%) and 2 patients a valve stenosis (10%). The mean EF was 49%. 16 patients underwent a mitral valve repair (72.7%) and 8 patients had a replacement (36.3%).

We did not see isolated mitral valve stenosis, but a combination of regurgitation and stenosis. So a rheumatic disease can be nearly excluded.

### Classification

We classified the patients in two groups with the histopathological finding of myxomatous degeneration of the tissue (Group I: Myxomatous mitral valve and group II: non myxomatous mitral valve). The tissue for the experimental set-up was taken from the right auricle before implementation of the extracorporal circulation. Ethic’s approval is available (According to § 14 AVB, Absatz 3: Patients, who will be admitted to the University Hospital will be informed and asked, if they agree to use tissue, that will be withdrawn within the operation, for research work. This tissue can be used without making further applications at the ethical review committee; http://www.laek-rlp.de).

### Pathological examination

The tissue was examined in the department of pathology. The tissue was microscopically examined by performing a haematoxylin-eosin staining.

### Experimental set-up

The tissue was transported in an oxygenated cardioplegic solution containing BDM (Butanedione-Monoxim, 30 mM) to the laboratory and prepared in a special way: skinned fibers were prepared while being incubated in a special solution containing 50% glycerol (Contents [mM]: Imidazole 20; Sodium azide 10; EGTA 4; Dithioerythriol 2; Magnesium chloride 5; ATP 5; ph 7) and subsequently stored in the same solution, containing 1% Triton-X-100 in addition, for 24 hours at 4°C. For our experimental approach we used skinned muscle stripes with a size of 2–2,5 mm × 0,3 mm, which were finally mounted on a force transducer. We took 3 fibers from each patient to perform the examination.

The approach with skinned fiber is well established in our laboratory. First described from Weber and Portzehl [16] it is a safe and reproducible method to remove cell-membrane dependent processes and to create an artificial experimental environment for examining the contractile apparatus. The fibers are first exposed to a so-called relaxation solution (Contents [mM]: Imidazole 20, Phosphocreatine 10; Sodium azide 10; EGTA 10; Magnesium chloride 25; Dithioerythriol 2; ATP 20 and 400 U/ml creatine kinase) and prestrechted to 20 mg. After achieving the force baseline, the experimental cycle starts. The calcium concentration starts at pCa 6.5 and increases stepwise to pCa 4.0 in the contraction solution, in which EGTA was substituted by CaEGTA. The desired calcium concentrations were calculated by a computer program, following the equation of Fabiato & Fabiato and given as pCa (-log of free [Ca]2++). The measured pCa-force values are recorded and collected on the attached computer (Scientific Instruments, Heidelberg, Germany).

### Statistical analysis

We performed the Wilcoxon rang sum test as a nonparametric test for non-normal distributions. Statistical significance was defined at a level of 5%.

## Results

**Figure 2:**
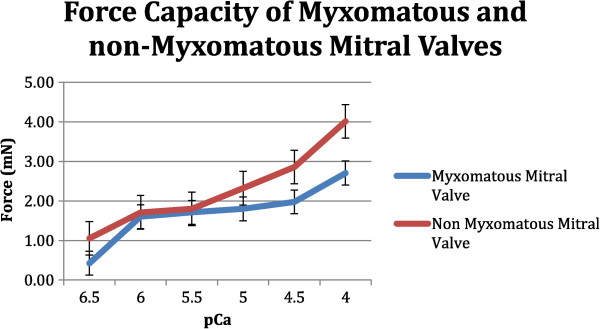
**Comparison of force capacities of myxomatous and non-myxomatous mitral valves.** Significant differences exist at pCa 4.0 (p 0.03) and 4.5 (p 0.04).

Patients without myxomatous mitral valve develop 4.0 mN ± 0.8 mN at the highest step of calcium concentration compared to 2.7 mN ± 0.4 mN of patients with myxomatous valve degeneration (Figure 2). This was significant less force in group I (p 0.03). When we compare the force values at the other 5 steps of calcium concentration we see significant differences in the higher calcium concentrations whereas the lower concentration do not show any significant difference: pCa 4.5 1.9 ± 0.8 mN (group I) versus 2.8 mN ± 1.2 mN (group II), p: 0.04 and at pCa 5.0: 1.8 mN ± 0.8 mN (group I) versus 2.3 mN ± 0.6 mN (group II), p: 0.05. At pCa step 5.5 group I achieves 1.7 mN ± 0.6 versus 1.8 ± 0.3 mN, at pCa 6.0 1.6 mN ± 0.4 versus 1.7 mN ± 0.3 and at the lowest concentration 0.4 mN ± 0.05 versus 1.05 mN ±0.2 mN. These force values were not significant different. But all force values of the non-myxomatous patients are higher compared to the myxomatous patients. The largest increase in force can be seen between pCa 4.5 and 4.0. According to that observation the calcium sensitivity of the myxomatous group is higher than in the non-myxomatous group. Calcium sensitivity was in group I at pCa 6.0 and in group II at pCa 5.

Comparing the clinical features we observed in these two groups, we find some noticeable results:

We observe a significant difference in ejection fraction (EF) among the groups: 49% in the non-myxomatous group versus 57% in the myxomatous group (p 0.03). Furthermore we observed significant higher incidence of diastolic dysfunction in the myxomatous group: 40% of the patients had a diastolic dysfunction (grade I-II). One patient even showed grade III. In group II 5 patients had diastolic dysfunction grade I-II (22,7%), this was significant (p 0.04). Six patients (24%) in group I had furthermore atrial fibrillation and 9 patients (38%) in group II, but this was not significant (p 0.4). When we have a look to the pathology of the patients we found significant more prolapses in the myxomatous group than in these patients without myxomatous mitral valve (p 0.02). Atrial dilatation occurs in 9 patients (36%), ventricular dilatation in 3 patients in group I (12%). An annulus dilatation appears in one patient (4%). In group II atrial dilatation emerges in 15 patients (68%), ventricular dilatation occurs in 3 patients (13%), annulus dilatation in 2 patients (9%). The appearance of atrial dilatation in the non-myxomatous group is significant (p 0.01). Comparing the surgical procedure of valve repair or replacement we observed a significant higher repair of mitral valve (p 0.02) in the non-myxomatous group (72%) compared to group I (48%). More than half of the patients in the myxomatous group underwent a replacement of the valve.

## Discussion

The myxomatous mitral valve degeneration has been studied for many years. As most common indication for mitral valve surgery due to severe regurgitation, the main interest so far was the histological and pathological investigation on the etiology of valve degeneration. Accordingly a variety of studies exist examining molecular pathways and the potential role of different molecules like the transforming growth factor superfamily or several enzymes [8, 13].

From surgical view we wanted to highlight another aspect of this valve disease: is the myxomatous degeneration a local process, taking place in the valves or has a combined effect on heart function? The right contractile property is important because right ventricular remodeling may play an important role in clinical outcome of patients undergoing mitral valve surgery [17, 18].

Our results show that atrial fibers from patients with myxomatous mitral valve degeneration achieve significant less force than those from patients without this pathological feature whereas calcium sensitivity is increased in the myxomatous mitral valve group. However the clinical findings of the myxomatous mitral valve group would assume a higher force curve than we found in this group because of younger age, less atrial as well as ventricular dilatation. In contrast patients in group II were older (67 years) and had a mean EF of 49%. Ninety% showed a mitral regurgitation associated with atrial dilatation (68%) and the majority was female (63%). The possible reasons are multifold.

It might reflect the affection of contractile capacity due to myxomatous degeneration: Brixius observed the same results in his studies with skinned fibers in failing and non-failing hearts of human donors and found higher calcium sensitivity in failing heart fibers with reduced force capacity [19]. Wankerl [20] furthermore examined different cardiac diseases with the skinned fiber model and found increased calcium sensitivity in atrial fibers in diseases with elevated right atrial pressure like in mitral valve regurgitation. And according to our results Arndt [21] furthermore observed in fibers from right atria of patients suffering from mitral valve disease increased calcium sensitivity and lower unloaded shortening velocity. This supports our assumption of compensating the reduced force with increased calcium sensitivity and gives some evidence for further impact on contractile behavior in MMVD.

Assuming that myxomatous mitral valve is not only a limited valve leaflet disease, we found some similarities with the patients examined by Matsumaru [22], who studied clinical and pathological differences of Carpentier’s differentiation of billowing mitral valve disease (BML) and fibroelastic deficiency (FED) and found that BML patients are more middle-aged, have a long term evolution of valve disease, multiple segments of billowing valve, more redundant tissue with a diffuse myxomatous infiltration, significant more dilated annulus and therefore require a multiple resection/suture technique. In opposite to that he found the FED patients to be older with focal myxomatous changes and restriction of thickening to the limited prolapsing segment. They mainly present no dilatation of the annulus and therefore need a less complicated surgical procedure. We share this observation of younger age in patients with a diffuse myxomatous degeneration and can support his finding of a preserved three-layer architecture of the leaflet tissue in the FED group, who are older, have more focal myxomatous changes and underwent more repair procedures than the younger patients in our myxomatous mitral valve group. Another evidence of impact of this pathological feature on surgical procedure gives Flameng [23]: he assessed echocardiographic studies including recurrence-free rate after valve repair in M. Barlow and reported an influence of etiology of myxomatous degeneration on complexity of plasty techniques [23]. An important observation from surgical view was made Adawil [24]: he gives some additional impressions to our assumption of the clinical picture consisting of older age, more recent symptoms and non myxomatous mitral valves: he found more calcific valves changes and signs of degeneration [24] and this is according to our results: we observed a more frequently valve stenosis component in this group which might give slight evidence for more annulus and perivalvular calcification. Another possible reason for reduced forces in the myxomatous group might also be the higher rate of diastolic dysfunction: There are several studies in literature, which assume that perturbations at the cellular level like impaired Ca 2+ - handling, extracellular matrix modification and myofilament dysfunction might cause diastolic dysfunction [25]. In our observation the diastolic dysfunction, patients was diagnosed in group I, can affect the force values, which actually were reduced. The incidence was higher in the myxomatous valve group and -as increasingly recognized in the past- diastolic dysfunction can emerge with a preserved left ventricular function, imaged as normal ejection fraction. Recent studies showed that 40-50% of patients even with "congestive heart failure" had an LVEF > 50% [26].

Another possible influencing factor could be the gender distribution: The higher number of females in group II might influence the force values: several studies about contractility and calcium sensitivity illustrate greater tension and higher calcium sensitivity [27, 28]. A gender dependent impact on the extent of mitral prolapse was demonstrated by Avierinos et al. [9], who found that female patients more often have anterior prolapse and more leaflet thickening and therefore lower regurgitation grades and higher ejection fraction. This may reflect the higher force in the non-myxomatous group with female majority (63%). So a gender related influence on the force values cannot be excluded.

## Conclusion

This study should highlights that myxomatous mitral valve is not only a pathological diagnosis but has some clinical aspects, which might influence the surgical therapy and result. In conclusion we observed an association of myxomatous mitral valve disease with reduced force capacity, increased calcium sensitivity and higher incidence of diastolic dysfunction. An early therapy of risc factors especially in patients with valve regurgitation (volume overload) could be made as a careful recommendation for clinical practice.

### Limitations

As mentioned above the variety of possible influencing factors on cardiac force development is broad. Of course the complexity of cardiac contraction cycle cannot be imaged in a single experimental approach, but it might give some evidence for an affection of myocardial apparatus. We are aware that we examine right atrial tissue and deduce our experimental observation to the right ventricle, but different studies [8, 29] prove a similar contractile behavior of atrial and ventricular myofilaments and studies exist about calcium sensitivity in atrial and ventricular skinned cardiac fibers in different cardiac diseases [20], so conclusions seem justified.

### Details of ethics approval

According to § 14 AVB, Absatz 3: Patients, who will be admitted to the University Hospital will be informed and asked, if they agree to use tissue, that will be withdrawn within the operation, for research work. This tissue can be used without making further applications at the ethical review committee. (See: http://www.laek-rlp.de).
